# The Value of the Evidence Integration Triangle

**DOI:** 10.1093/geroni/igab046.1322

**Published:** 2021-12-17

**Authors:** Barbara Resnick

**Affiliations:** University of Maryland School of Nursing, Baltimore, Maryland, United States

## Abstract

The Evidence Integration Triangle involved engaging stakeholders in the 12 month FFC-AL-EIT activities including identifying community specific goals, supporting the staff implementing the intervention, and intervening when champions or staff were not engaged in intervention activities. Ongoing participation of the stakeholder team occurred through monthly meetings. Evaluation of implementation was based on the Reach Effectiveness Adoption Implementation and Maintenance (RE-AIM) Model. Reach was based on 85 of 90 communities participating and 794 residents recruited. Effectiveness was supported based on less functional decline and more function focused care performed by residents. Adoption was based on evidence that monthly meetings were held, 77% of settings engaged as, or more than expected, and caregivers increased the amount of function focused care provided. The intervention was implemented as intended, knowledge was received, and environments and policies supporting function focused care were maintained. The Evidence Integration Triangle is an effective implementation approach for assisted living.

